# Biologging assessment of behavioural and physiological responses of European seabass (*Dicentrarchus labrax*) during stress challenges

**DOI:** 10.1038/s41598-025-26039-4

**Published:** 2025-11-26

**Authors:** Esther Hoyo-Alvarez, Joaquim Tomàs-Ferrer, Martin J. Lankheet, Wout Abbink, Arjan P. Palstra, Pablo Arechavala-Lopez

**Affiliations:** 1 Institut Mediterrani d’Estudis Avançats (IMEDEA-CSIC/UIB), 07190 Esporles, Mallorca Spain; 2 Laboratori d’Investigacions Marines i Aqüicultura (LIMIA-IRFAP), IRFAP, CAIB, Unitat Associada al CSIC per IMEDEA, 07157 Port d’Andratx, Mallorca Spain; 3 Experimental Zoology Group, Wageningen University & Research, Wageningen, 6700 AH The Netherlands; 4 Animal Breeding and Genomics, Wageningen University & Research, Wageningen, 6700 AH The Netherlands

**Keywords:** Swimming performance, Crowding, Cardiac response, Stress response, Biologging, Aquaculture, Animal behaviour, Animal physiology, Ichthyology

## Abstract

Stress significantly impacts fish welfare, and for a comprehensive evaluation, welfare assessment requires an integrative approach. The objective of this study is to gain insight into the physiological and behavioural responses of European sea bass subjected to swimming and crowding stress tests through biologging. Individuals implanted with biologgers were subjected to swim tunnel and crowding tests, measuring locomotion, oxygen consumption, heart rate, acceleration and QRS-wave amplitude. During swimming stress tests, oxygen consumption correlated positively with heart rate (R^2^ = 0.56, *p* < 0.001) and acceleration (R^2^ = 0.76, *p* < 0.001). Acceleration values recorded by biologgers were strongly correlated with head and tail beat frequency (R^2^ = 0.69 and R2 = 0.70 respectively; *p* < 0.001), validating heart rate and acceleration as reliable proxies for energy expenditure in sea bass. During the crowding challenge, heart rate increased progressively with each stressing event, while QRS-wave amplitude and acceleration peaked with stress but decreased in-between stressors. The assessment of physiological and behavioural responses of sea bass to swimming and crowding stress tests with biologgers allows the characterization of four welfare states, and therefore, highlights the potential of biologging for fish stress response and welfare monitoring.

## Introduction

Assessing fish welfare is complex and requires an integrative approach, encompassing physiology, behaviour and biological performance^1,2^. A comprehensive understanding of the basic biology of fish species is crucial for livestock production to enhance welfare and productivity in aquaculture^3^. Fish welfare is significantly impacted by stress, with plasma cortisol and secondary stress response indicators such as blood glucose and lactate levels serving as welfare indicators^4^. While acute stress responses may be adaptive, chronic or repeated stress can compromise welfare^5,6^. This is due to the sustained allostatic load and energy demand associated with prolonged stress, which can impair growth, immune function, and reproductive capacity^5^. Additionally, chronic stress is associated with behavioural alterations such as reduced feeding, impaired learning, and increased refuge use, suggesting a negative affective state in the fish^6^. Although stress indicators are useful, they may be insufficient on their own for evaluating welfare^7–9^. Therefore, they should be complemented by other measures for comprehensive welfare assessment^7^.

Innovative approaches using spontaneous swimming performance, cardiac activity, and behaviour have been developed as proxies for fish welfare and energy use^10–12^. The energy economy of fish can be evaluated through swim-fitness and crowding stress challenge tests, which measure aerobic and anaerobic metabolic components^13^. Swimming performance and the use of aerobic/anaerobic metabolism are key factors in assessing the physiological state of fish and their ability to cope with stressors^14^.

A swim-fitness test generally involves swimming fish at increasing speeds in a swim tunnel^3,15–17^. The duration of the test and the flow speed determine the aerobic and anaerobic components of the metabolic performance, reflecting the contributions of the red and white skeletal muscles, respectively^13^. Measuring oxygen consumption during swim-fitness tests enables quantification of the aerobic component, and locomotory behaviour analysis can explain variations in oxygen consumption^17,18^. Locomotion occurs when animals spend energy to contract muscles, leading to body acceleration. Accurate measurement of animal acceleration is a good proxy for energy expenditure during activity^3,19,20^. Locomotor performance and associated metabolic costs are often coupled with life history traits, involving trade-offs related to growth and energy expenditure^21,22^.

Heart rate and acceleration are often strongly correlated with oxygen consumption rate and locomotory parameters^23,24^. Palstra et al.^17^ conducted swim-fitness tests and crowding challenge tests in Yellowtail kingfish (*Seriola lalandi*) and reported a linear increase in oxygen consumption and heart rate with swimming speed, reflected by locomotory parameters such as tail beat frequency. Although the impact of heart rate on oxygen consumption during exercise might be minimal, in some species such as salmonids, stroke volume and the efficiency of oxygen extraction could be more significant factors^25^. The capacity to increase stroke volume varies within species, leading to different levels of heart rate increase in response to exercise^26^. Stroke volume is related to the amplitude of the QRS wave, although it is not a direct relationship^25,27^. Additionally, several factors such as surgical placement of the tag, muscular tissue mass and tissue conductivity can influence the relationship between stroke volume and amplitude of the QRS wave^28,29^.

Recently, precision tools such as acoustic telemetry and biologger devices have been used to monitor fish physiology and behaviour, enhancing the understanding of basic biology and the evaluation of fish welfare during livestock production^2,30^. In particular, biologgers have the potential to provide continuous monitoring of fish, both in terms of internal state (e.g. heart rate) and behaviour (e.g. swimming activity)^29,31^, allowing estimations of energy use and assessment of stress responses^17,32,33^. Biologging devices have been shown to be effective in monitoring activity and heart rate in several species such as Atlantic salmon, Rainbow trout, Yellowtail kingfish, and Gilthead seabream among others^17,32–35^. Some studies have assessed the relationship between acceleration, heart rate and locomotory parameters with energy expenditure, but the majority of them are focused on salmonid species (e.g.^20,23,33^).

European sea bass (*Dicentrarchus labrax*) is one of the most important farmed fish species in Mediterranean aquaculture^36^. Despite this, there are few studies assessing the relationship between acceleration and body motion in sea bass^37–39^, but there are no studies assessing correlations between cardiac activity and oxygen consumption for this species^7^. Although biologger measurements can be used to assess behavioural and physiological parameters, lab-based calibrations between biologger output and oxygen consumption are necessary to provide accurate estimates of energy use in European sea bass^3,40^.

Therefore, the aim of this study is to provide a comprehensive understanding of the evaluation of welfare states of European sea bass by assessing the behavioural and physiological response through implanted biologgers at increasing swimming speeds and during crowding stress challenge tests.

## Results

### Swimming stress challenge test

Significant differences in standard length (SL) and body weight (BW) were found between control and tagged fish, as the larger fish needed to be selected for surgery (SL: ANOVA, *p* < 0.01; BW: Kruskal-Wallis, *p* < 0.001) (Table 1). The optimal swimming speed (U_opt_) did not differ between groups (U_opt_*=* 0.74 m.s^− 1^; Kruskal-Wallis, *p* > 0.05). However, significant differences were found in the minimum cost of transport (COT_min_), with COT_min_ = 85 ± 2 mg.kg^− 1^.km^− 1^ for the control group and COT_min_=113 ± 11 mg.kg^− 1^.km^− 1^ for the tagged group (ANOVA, *p* < 0.01) (Table 1).

**Table 1:** Mean values (± SE) of body length (BL), body weight (BW), optimal swimming speed (Uopt) and minimum cost of transport (COT_min_) for tagged and non-tagged sea bass in the swim-tunnels, and significance of differences between tagged (*N* = 12) and non-tagged fish (*N* = 6) for all parameters (significant differences are marked with an asterisk).

	All	Tagged	Control	*p*	Test
SL (cm)	28.3 ± 0.4	29.1 ± 0.5	26.8 ± 0.5	0.0085*	a
BW (g)	391 ± 14	423 ± 13	327 ± 12	0.0007*	b
U_opt_ (m.s^− 1^)	0.74 ± 0.014	0.74 ± 0.014	0.74 ± 0.036	0.925	b
U_opt_ (bl.s^− 1^)	2.62 ± 0.07	2.55 ± 0.07	2.75 ± 0.14	0.190	b
COT_min_(mg.kg^− 1^.km^− 1^)	95 ± 5	85 ± 2	113 ± 11	0.0032*	a

*Test: a = ANOVA; b = Kruskal-Wallis*.

**Fig. 1:**
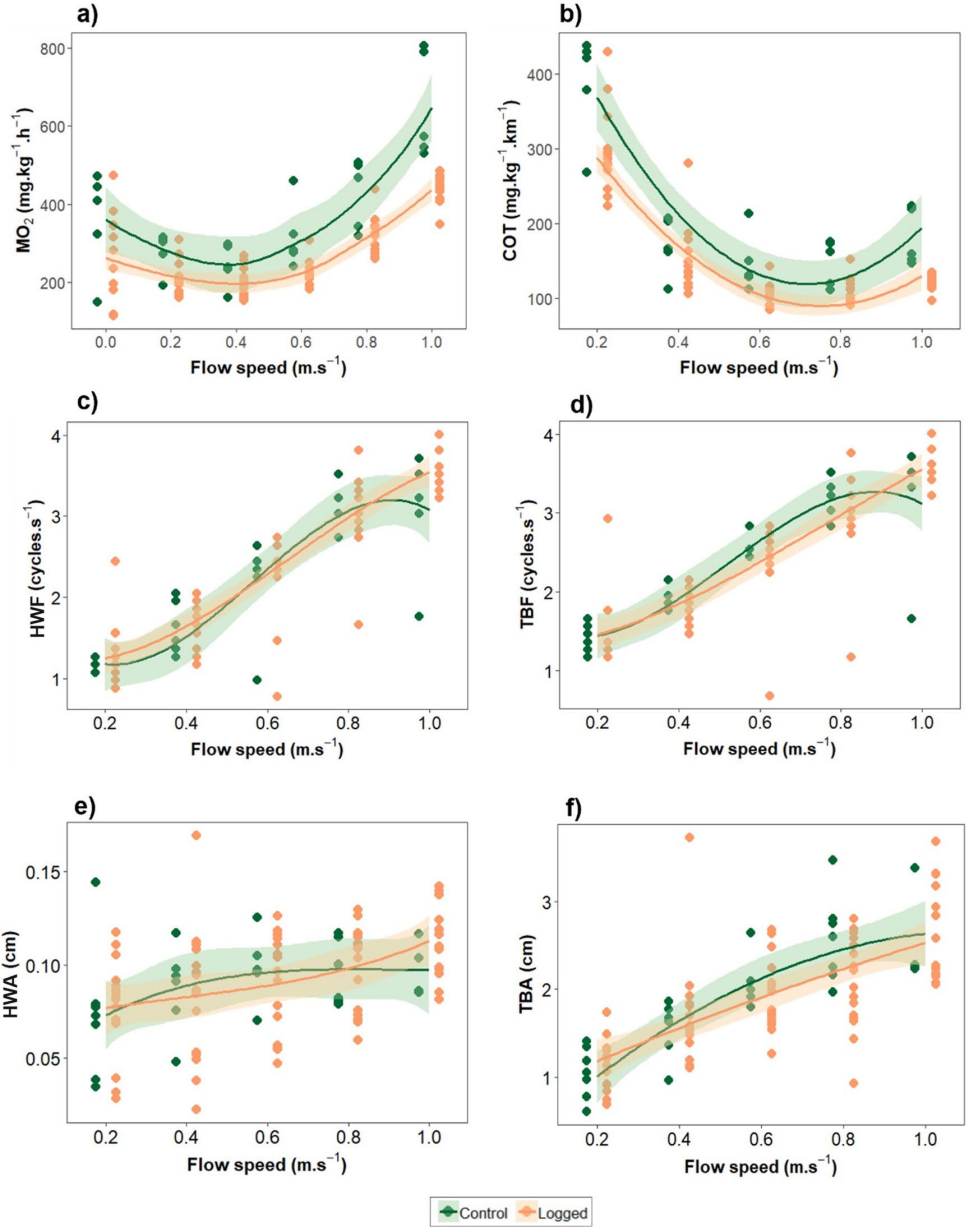
Respirometry and motion parameters during the swimming stress challenge of tagged (orange) and non-tagged (green) individuals of sea bass. **(a)** Oxygen consumption (MO_2_); **(b)** Cost of transport (COT); **(c)** Head width frequency (HWF); **(d)** Tail beat frequency (TBF); **(e)** Head width amplitude (HWA); **(f)** Tail beat amplitude (TBA). Data points have been slightly displaced along the X-axis to avoid overlap and improve visual clarity.

To better assess the non-linear relationship between respirometry and motion parameters with swimming speed, polynomial linear models were adjusted. None of the individuals reached critical speed during the swimming challenge. Both tagged and non-tagged groups followed the same relationship pattern between oxygen consumption (MO_2_) and swimming speed (R^2^_control_ = 0.46, R^2^_tagged_ = 0.46). However, MO_2_ at the highest swimming speed (1 m.s^− 1^) was found to be significantly lower in tagged fish compared to non-tagged fish (ANOVA and TukeyHSD, *p* < 0.0001) (Fig. 1a). Regarding COT, both groups followed a similar trend, showing higher values at low flow speeds and reaching the minimum COT value at 0.74 m.s ^− 1^ (R^2^_control_ = 0.77, R^2^_tagged_ = 0.80). At the lowest speed (0.2 m.s^− 1^), non-tagged fish had a significantly higher COT than tagged fish (ANOVA and TukeyHSD, *p* < 0.001) (Fig. 1b).

The fitted models revealed a significant positive correlation of swimming speed with head frequency (HWF; R^2^_control_ = 0.78, R^2^_tagged_ = 0.84) and tail beat frequency (TBF: R^2^_control_ = 0.80, R^2^_tagged_ = 0.78), both increasing with flow speed (LM, *p* < 0.01 in both cases) (Fig. 1c, d). The amplitude of the head and tail beats showed a lower positive correlation (HWA: R^2^_control_ = 0.14; R^2^_tagged_ = 0.18; TBA: R^2^_control_ = 0.71; R^2^_tagged_ = 0.18; Fig. 1e, f). No significant differences were found in the locomotion parameters between tagged and non-tagged fish at each flow speed (ANOVA and TukeyHSD, *p* > 0.05 in all cases).

**Fig. 2:**
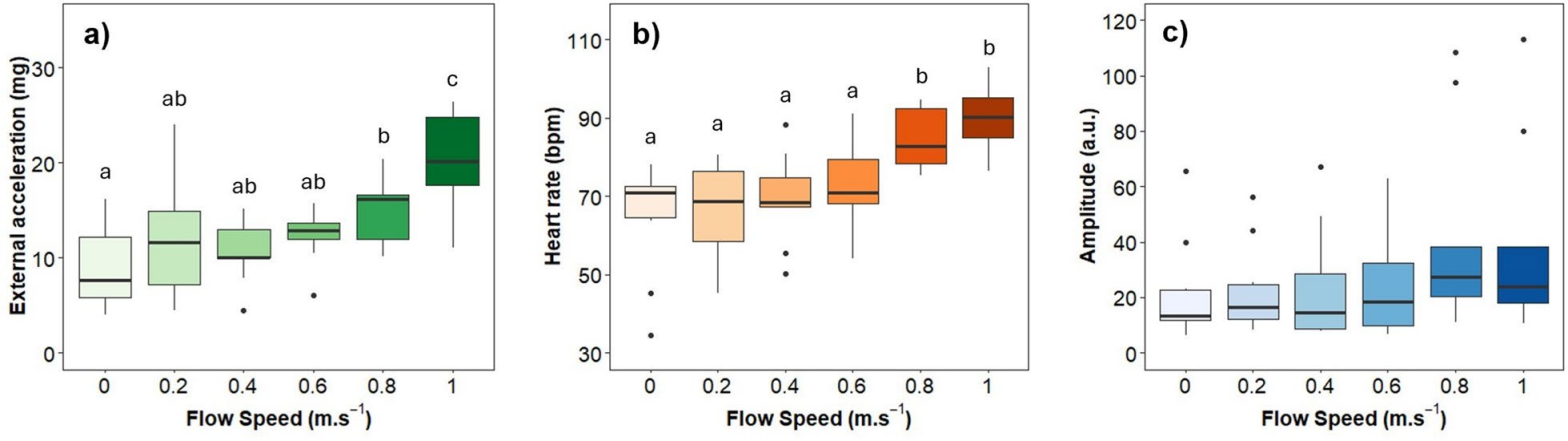
Cardiac activity measured by biologgers during the swimming stress challenge test. **(a)** External acceleration (ACC); **(b)** Heart rate (HR); **(c)** Amplitude of the QRS wave (AMP).

**Fig. 3:**
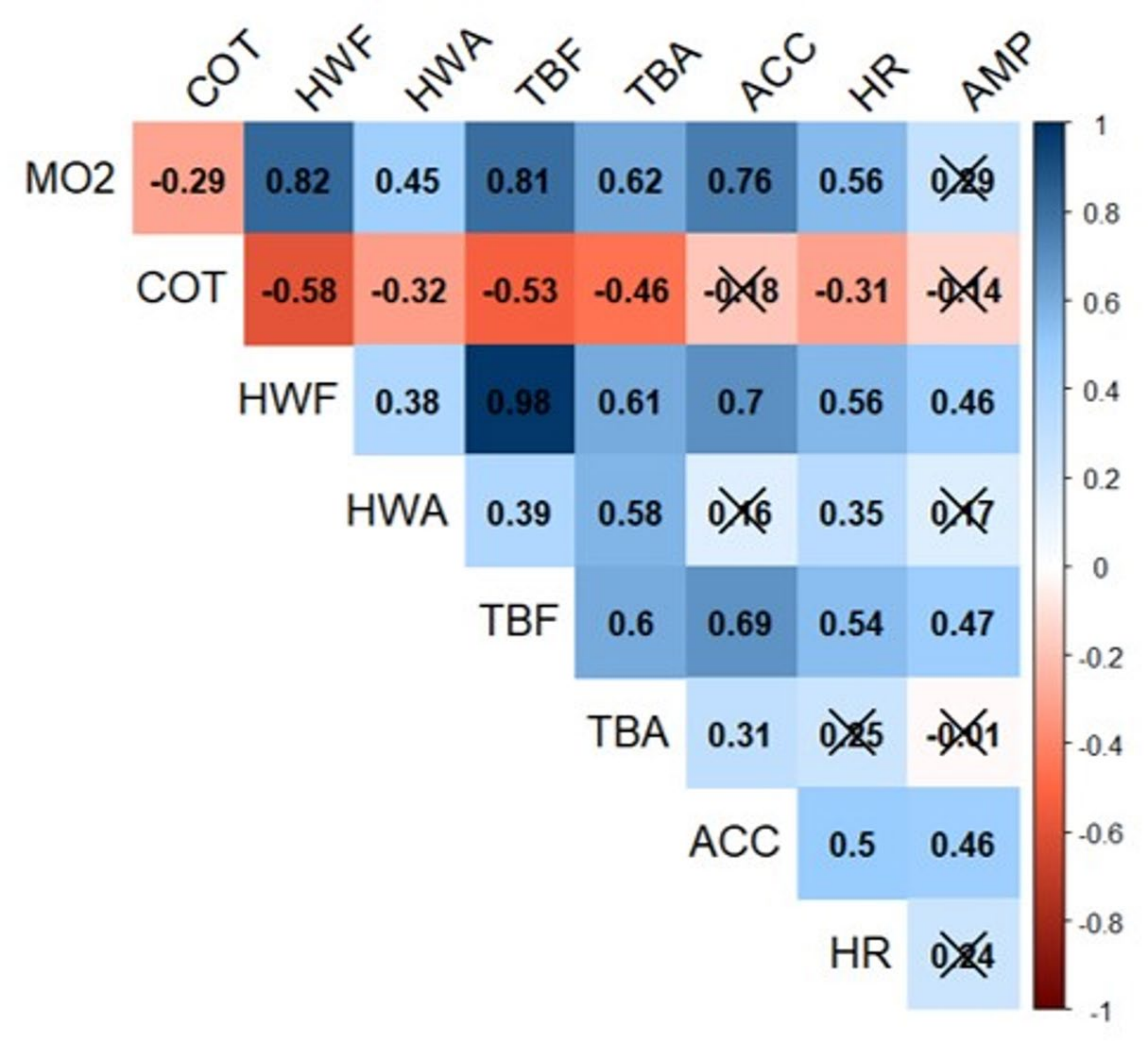
Correlation matrix and determination coefficient (R^2^) between cost of transport (COT), oxygen consumption (MO2), head width frequency (HWF), head width amplitude (HWA), tail beat frequency (TBF), tail beat amplitude (TBA), Acceleration (ACC), heart rate (HR) and amplitude of the QRS wave (AMP). Crosses indicate no significant correlation between the parameters in question (LM, *p* > 0.05).

Seabass external accelerations (ACC) showed a significant positive correlation with flow speed (exponential fit: R^2^ = 0.45, *p* < 0.0001) (Fig. 2a). Comparing different flow speeds, the first four speeds (0.2–0.8 m.s^− 1^) showed no significant differences in ACC among them (LMM, *p* > 0.05), with the lowest variability observed at 0.6 m.s ^− 1^. However, there is an exponential increase in ACC at the highest speed (1 m.s^− 1^), which is significantly greater than the ACC in the other speeds (LMM, *p* < 0.001). Regarding cardiac activity, the HR also followed a positive trend (exponential fit: R^2^ = 0.23, *p <* 0.0001) (Fig. 2b). Similar to ACC, the first four speeds exhibit no significant differences on HR among them (LMM, *p >* 0.05). Once the optimal swimming speed (U_opt_) of 0.74 m.s^− 1^ is surpassed, the HR at higher speeds (0.8 and 1 m.s^− 1^) becomes significantly higher as compared to the lower speeds (LMM, *p* < 0.05 and *p* < 0.001 respectively) (Fig. 2b). There is virtually no correlation between the amplitude (AMP) of the cardiac signal and swimming speed (R^2^ = 0.07) (Fig. 2c). Additionally, there are no significant differences in the mean AMP values across different swimming speeds (LMM, *p* > 0.05). A significant negative correlation was observed between MO_2_ and COT (R^2^ = -0.29, *p* < 0.05) (Fig. 3). Among the motion parameters, strong correlations were detected: HWF vs. TBF (R^2^ = 0.98), TBF vs. TBA (R^2^ = 0.60), HWF vs. HWA (R^2^ = 0.38) and TBA vs. HWA (R^2^ = 0.58) (*p* < 0.05 in all cases) (Fig. 3). Given the stronger positive correlations between TBF and other motion parameters, and the importance of MO_2_ as a physiological measure, MO_2_ and TBF were selected as the primary variables for further analysis.

**Fig. 4:**
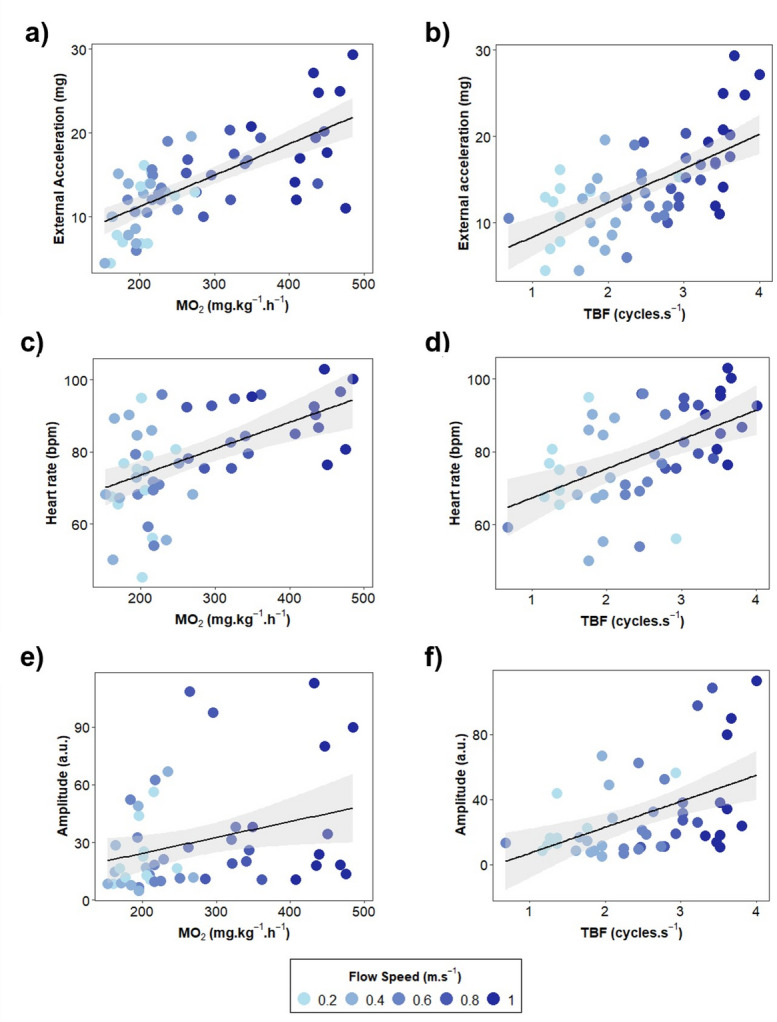
Correlations between biologger measurements and respirometry parameters during the swimming stress challenge test. **(a)** Acceleration (ACC) vs. Oxygen consumption (MO_2_); **(b)** Acceleration (ACC) vs. Tail beat frequency (TBF); **(c)** Heart rate (HR) vs. Oxygen consumption (MO_2_); **(d)** Heart rate (HR) vs. Tail beat frequency (TBF); **(e)** Amplitude (AMP) vs. Oxygen consumption (MO_2_); **(f)** Amplitude (AMP) vs. Tail beat frequency (TBF).

ACC showed a strong positive correlation with MO_2_ and TBF (R^2^ = 0.76 and R^2^ = 0.69, *p* < 0.001) (Figs. 3 and 4a and b). Similarly, HR was significantly correlated with MO_2_ (R^2^ = 0.56, *p* < 0.001) and TBF (R^2^ = 0.54, *p <* 0.01) (Figs. 3 and 4c and d). Although the correlation between the AMP and MO_2_ was not significant (R^2^ = 0.29, *p* > 0.05), both parameters tended to increase at higher flow speeds (Figs. 3 and 4e). The relationship between AMP and TBF followed a significant positive correlation (R^2^ = 0.47, *p* < 0.05) (Figs. 3 and 4f).

### Crowding stress challenge test

**Fig. 5:**
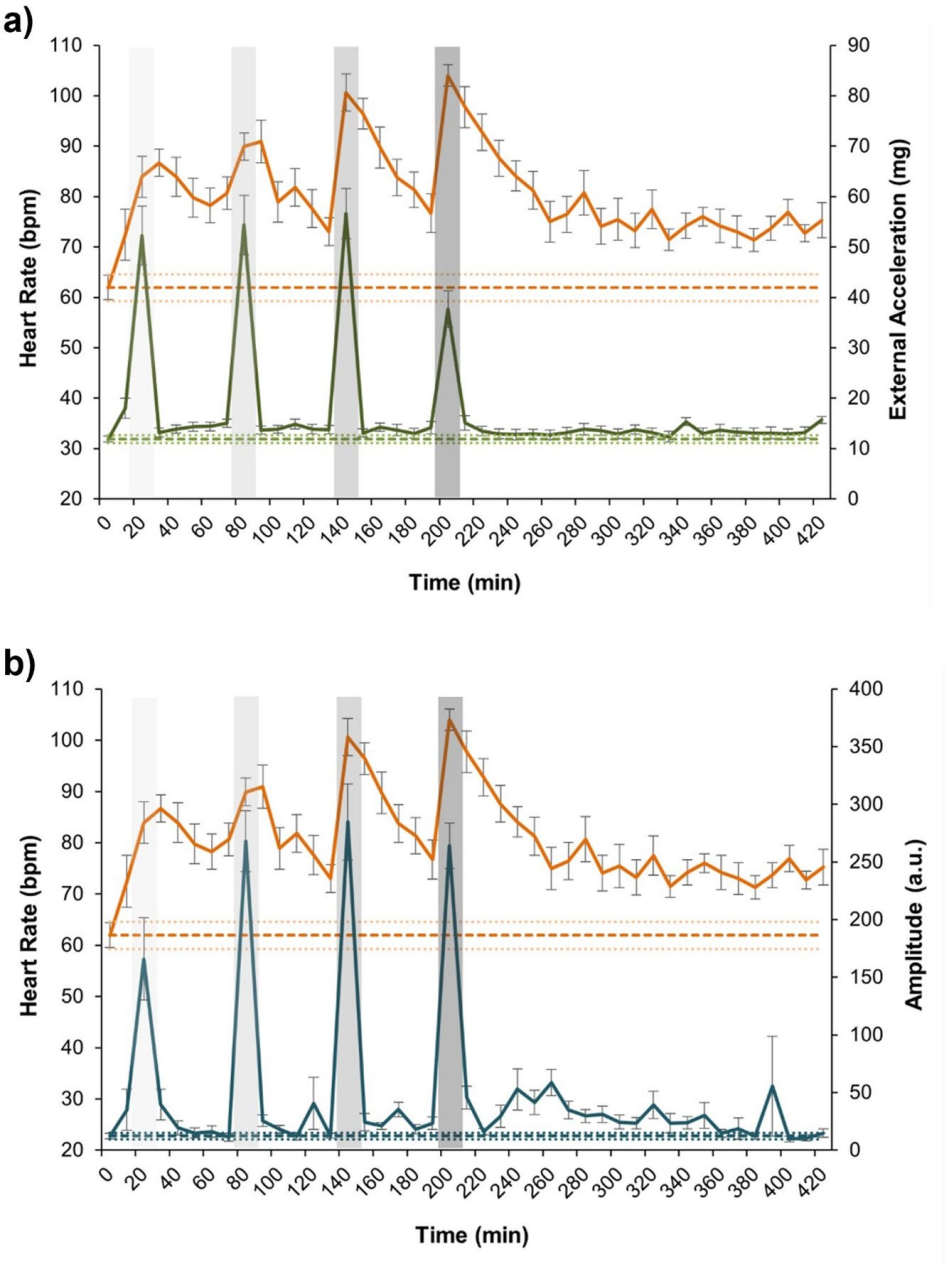
Heart rate (HR), acceleration (ACC) and amplitude of QRS wave (AMP) during the crowding stress challenge test. **(a)** Heart rate values (Orange) and Acceleration (green); **(b)** Heart rate values (Orange) and Amplitude of the QRS wave (Blue). Mean basal values of each variable are shown in dotted lines (± SE). Shaded zones correspond to the subsequent crowding stress induction steps (1 to 4).

**Fig. 6:**
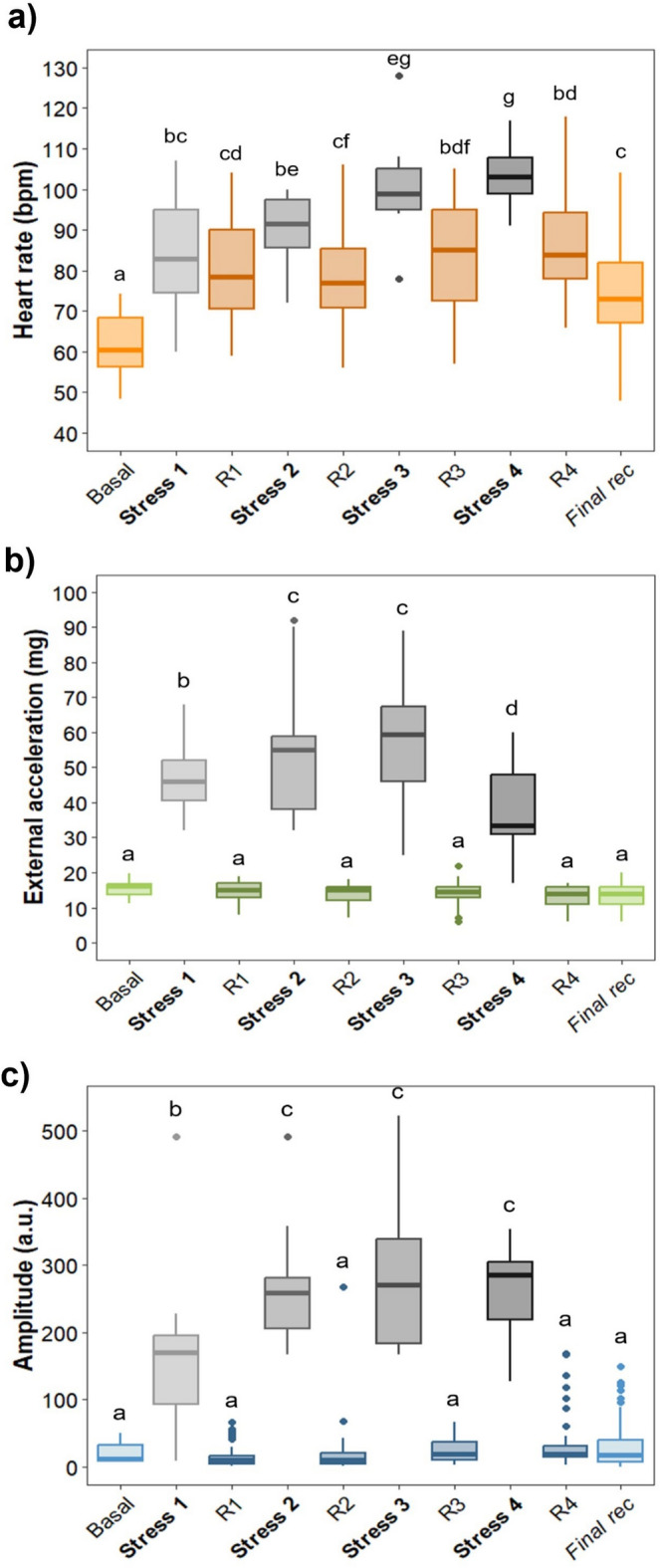
Cardiac response to the crowding stress challenge test. Baseline values of each variable are shown on the *x* axis, as well as crowding stress induction steps (Stress 1- Stress 4), recovery period between stressors (R1 - R4) and values 2 to 4 h after the end of the crowding stress challenge test (*Final rec*). Different letters mean significant differences (LMM, *p* < 0.05). **(a)** Heart rate (HR); **(b)** Acceleration (ACC); **(c)** Amplitude of the QRS wave (AMP).

Seabass exposed to the crowding stress challenge showed progressively increasing HR which each subsequent stress event (Fig. 5). HR increased smoothly during the first two crowding events (HR_S1_ = 83.91 ± 4.07 bpm; HR_S2_ = 89.9 ± 2.96 bpm), but the increase was more rapid in the last two events, reaching its maximum at the fourth event (HR_S4_ = 104 ± 2.10 bpm). Within each stress event, HR values did not significantly differ from the preceding event (LMM; *p >* 0.05) but were significantly different across all the other events (LMM; *p* ≤ 0.01 in all cases) (Fig. 6a). Between stress events, HR decreased gradually but did not return to basal levels (HR_basal_ = 61.94 ± 2.66 bpm). After the crowding stress challenge, HR did not show a complete recovery, remaining significantly elevated three hours later compared to basal levels (HR_post_ = 76.99 ± 3.16 bpm; LMM; *p* < 0.01) (Figs. 5 and 6a). ACC showed sharp peaks coinciding with crowding stress events (Fig. 5a). High increases in ACC were observed during the first three events (ACC_S1_ = 52.25 ± 5.83 mg; ACC_S2_ = 54.33 ± 5.87 mg; ACC_S3_ = 56.58 ± 4.99 mg), with a notable decrease during the fourth event compared to preceding events (ACC_S4_ = 37.66 ± 3.61 mg; LMM; *p* < 0.001 in all cases) (Fig. 6b). All the reported values for ACC during the stress events (S1 – S4) resulted to be significantly higher than recovery and basal values (LMM; *p* < 0.001 in all cases). After each stress event, ACC values returned to basal levels, including three hours post-stress when reported values were similar to basal levels (ACC_post_ = 13.35 ± 0.97 mg; ACC_basal_ = 11.83 ± 0.79 mg; LMM; *p >* 0.05 in all cases) (Figs. 5a and 6b). High peaks in AMP were also recorded during the crowding stress events (AMP_S1_ = 165.85 ± 35.60 a.u.; AMP_S2_ = 267.83 ± 27.56 a.u.; AMP_S3_ = 284.63 ± 34.53 a.u.; AMP_S4_ = 264.04 ± 19.81 a.u.). AMP during S2-S4 was significantly higher than during the first stress event (LMM; *p* < 0.01 in all cases) (Fig. 6c). Significant differences were also observed between AMP during stress events and that during the recovery periods (LMM; *p* < 0.01 in all cases). Following the crowding stress challenge test, AMP values returned to basal levels (AMP_post_ = 14.88 ± 3.53 a.u.; AMP_basal_ = 12.24 ± 2.72 a.u.; LMM; *p* > 0.05) (Figs. 5b and 6c).

## Discussion

This study successfully described the physiological and behavioural responses of European sea bass to swimming and crowding stress challenges, revealing temporal patterns and correlations among studied parameters.

Individuals did not reach the critical speed, as the maximum tested flow speed was 1 m.s^− 1^ due to technical limitations, and the reported critical speed for sea bass is 1.32 ± 0.11 m.s^− 1^^7^. Moreover, the primary objective of the study was the calculation of U_opt_ and COT_min_. At higher speeds, tagged individuals exhibited significantly lower MO_2_ rates, which is likely due to the enhanced swimming efficiency associated with larger fish size^14^. The analysis of body motion parameters revealed no statistically significant differences between groups, which linearly increased with water speed, indicating that tag implantation does not have any adverse effects on fish locomotion. Therefore, the reported differences in COT_min_ and MO_2_ between the groups may be attributed to size variations rather than the effects of tag implantation, as smaller individuals are generally known to exhibit higher mass-specific oxygen consumption rates, which decrease logarithmically with body size^41^. However, although no significant differences were observed in locomotory parameters between control and tagged fish, it cannot be entirely ruled out the possibility of subtle effects related to tag implantation. Notably, MO_2_ and COT followed a quadratic relationship with swimming speed, showing values between ≈ 200 and 800 mg.kg^− 1^.h^− 1^ and ≈ 450 and 100 mg.kg^− 1^.km^− 1^ respectively, which are consistent with those previously reported by Zupa et al.^14^.

The ACC values obtained from seabass with the implanted biologgers showed an increase with flow speed, exhibiting a pattern comparable to that previously observed for MO_2_ Carbonara et al.^7^ conducted a similar experiment with sea bass utilising acoustic transmitters to assess swimming activity in swim tunnels. The authors reported an exponential increase in swimming activity with flow speed, comparable to the values obtained by biologgers in this study. Furthermore, the locomotion parameters were strongly correlated with the ACC values obtained by the surgically implanted biologgers, thereby demonstrating that Star-Oddi biologgers provide reliable estimates of fish swimming activity and locomotion. It can be therefore concluded that data obtained from biologgers may be used as a measure of swimming behaviour in captivity, and therefore as an indicator of fish welfare. Moreover, the strong correlation between ACC and MO_2_ (R^2^ = 0.76, *p* < 0.001) or ACC and TBF (R^2^ = 0.81, *p* < 0.001) during swim challenge indicates that biologgers and locomotion can be used to extrapolate MO_2_ in future studies. However, it is important to note that forced swimming trials may not fully represent natural swimming conditions as the possible stress state induced by forced swimming challenges may lead to an overestimation of oxygen consumption^7^. Therefore, biologging and locomotion monitoring on free-swimming fish could provide more reliable insight into their swimming activity and behavioural responses of sea bass in natural or aquaculture environments^35^.

A significant increase in HR can be observed when the optimal swimming speed (U_opt_ = 0.74 m.s ^− 1^) is exceeded and when swimming activity is required to be above the regular range. The resulting positive correlation between HR and MO_2_ (R^2^ = 0.56, *p <* 0.001) allows for the deduction of the functional relation of extracting oxygen from the water to the blood and then pumping it by the heart throughout the body. However, while a robust correlation can be established between these parameters, the conditions under which the relationship holds may be quite restricted. This is due to the fact that physiological states and environmental factors significantly influence the relationship between HR and MO_2_ such that multiple curves may exist for a single species^42^. As no studies have yet been published characterising this relationship using implanted biologgers in sea bass, direct comparisons are challenging and further research in this area is needed.

In addition to HR and oxygen extraction from blood, the stroke volume also contributes to the increase in oxygen consumption in fish^42^. The results demonstrated no significant variation in AMP during the swim challenge test, although inter-individual differences increased with increasing swimming speed. These results suggest that individuals may adopt different strategies to cope with exercise. However, in general terms, European sea bass primarily respond to sustained swimming by increasing HR, while the amplitude of the QRS complex remains stable. This pattern reflects the recruitment dynamics of red and white musculature in sea bass, where aerobic metabolism predominates at moderate speeds, and anaerobic metabolism is increasingly recruited as speed approaches critical thresholds^37,39^. This contrasts with findings in salmonids^26^, where increases in stroke volume, reflected by AMP, contribute significantly to the cardiac response during exercise. The present data indicate that, in sea bass, stroke volume may play a less prominent role under these conditions, with the cardiovascular response relying more strongly on changes in HR. Nonetheless, as the relationship between AMP and stroke volume is not direct, further research is needed to confirm this interpretation and better understand interspecies differences in cardiac regulation^25,27^.

During the crowding stress challenge test, the ACC values showed elevated peaks coinciding with the four crowding events. Following each event, the ACC returned to its basal levels. The ACC peak corresponding to the fourth stressing induction step was significantly lower than the one observed in the preceding steps. Palstra et al.^17^ observed a decline in ACC with each subsequent event when subjecting Yellowtail kingfish to the identical crowding stress challenge test, indicating that in this species, predictability reduces the stress response. However, in the case of European sea bass, the reduction in ACC during the last stressing event coincides with the maximum value of HR reported throughout the entire crowding stress challenge, indicating that it may correspond to a reactive response of the species (i.e. freezing behaviour). There was also a significant increase in HR values of seabass during the crowding stress challenge test. In between stressing events, HR did not return to its basal levels and in fact, it increased in each subsequent stress induction step, suggesting a cumulative effect on the cardiac response to the stressors. The amplitude of the QRS wave (AMP) increased abruptly in each stressing induction step, particularly in the three final events. These findings suggest that when facing an acute stress, sea bass tends to maximize oxygen extraction by regulating the stroke volume, which is reflected in the observed increase in the AMP detected by the implanted biologgers. After the end of each crowding stress event, maximization of oxygen extraction might be sustained primarily by an increase in HR rather than by an increase in stroke volume, inducing a reactive response. These findings differ from those reported by Bloecher et al.^31^, who evaluated cardiac response in Atlantic salmon subjected to crowding events and observed a decrease in heart rate associated with an increase in stroke volume. This mechanism is attributed to the great capacity of salmonids to increase stroke volume and evidences the variability of mechanisms to maximize cardiac output between fish species. In the case of European sea bass, an increase in the AMP may play a pivotal role when facing acute stressing events, while when stressors are sustained or chronic, increasing the HR might be the main mechanism to regulate cardiac output.

**Fig. 7:**
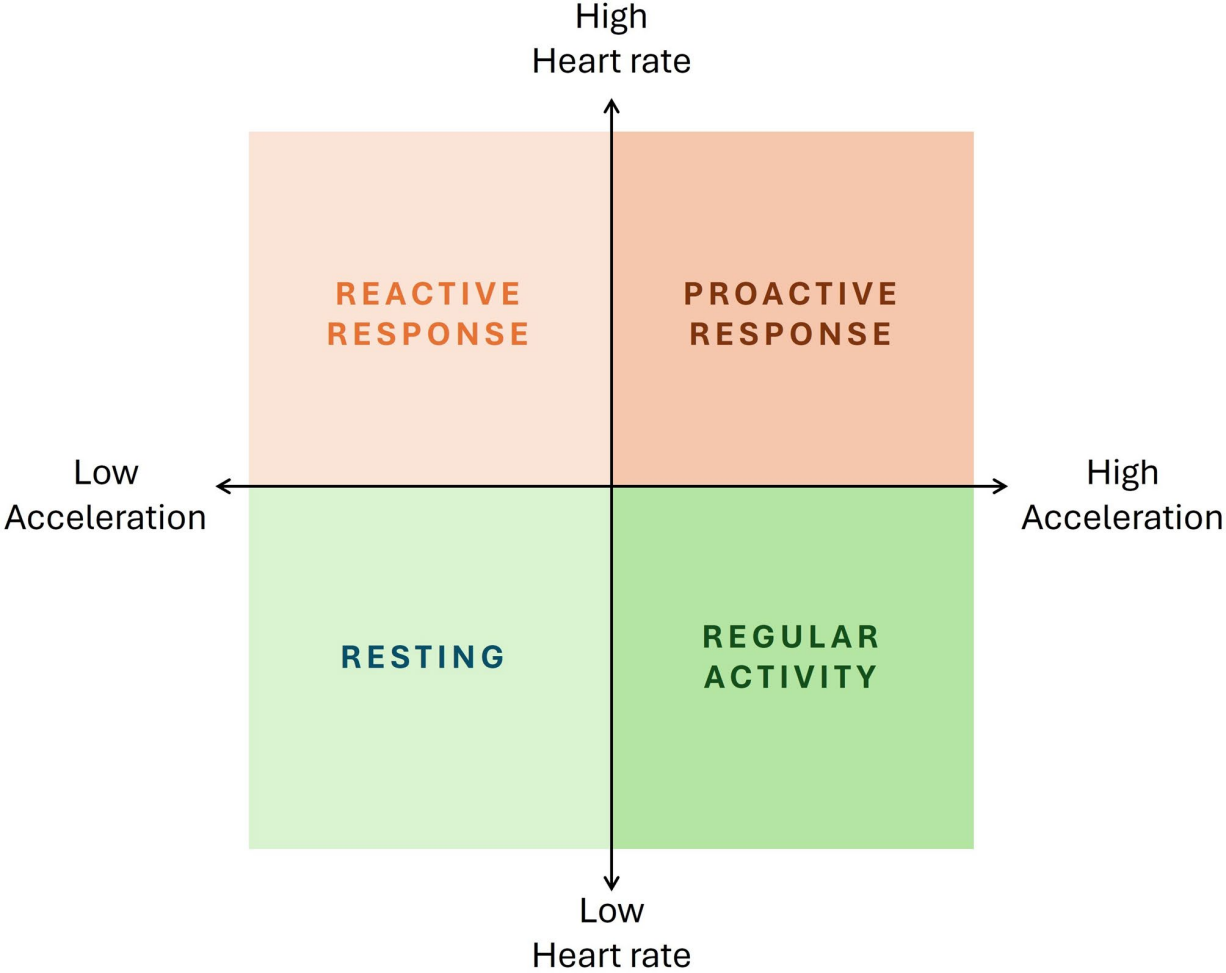
Behavioural states of European sea bass based on data obtained by implanted biologgers on captive individuals.

According to the definition of welfare proposed by Broom (1991), which describes it as “The state of the individual as it copes with the environment”, and based on the relationships between oxygen consumption, swimming activity and the cardiac response of European sea bass to swimming and crowding stress challenges observed in the present study, four distinct “fish welfare individual states” were characterized (Fig. 7), enhancing the interpretation of data obtained from implanted biologgers in future studies. First, “fish resting state” is characterized by low swimming activity and accelerations, low heart rate and low oxygen consumption. Second, “fish regular activity” involves moderate to elevated acceleration (diel movement patterns), but low heart rate and, consequently, low oxygen consumption. Third, a “fish reactive response” can be identified by low swimming activity and/or acceleration (e.g. freezing behaviour) and low amplitude of the QRS wave coupled with an elevated heart rate, which implies a high oxygen consumption rate. Lastly, a “fish proactive response” is associated with high levels of heart rate, high amplitude of the QRS wave and high oxygen consumption, which reflects an increase in oxygen demand. It is important to note that these welfare states are not discrete categories but rather exist on a continuum, with possible intermediate states that reflect varying degrees of physiological and behavioural responses, with different levels and durations in time. Moreover, the observed responses do not necessarily imply a negative stress state but rather may represent adaptive physiological and behavioural strategies to actively cope with environmental challenges.

This study has successfully characterized the behavioural and physiological responses of European sea bass under swimming and crowding stress challenges. The findings demonstrate the value of biologgers in analysing the correlation between swimming activity, cardiac response, oxygen consumption and locomotion parameters, and how these parameters can be interpreted to assess and determine the welfare state of a fish. Nevertheless, further research is required to gain a deeper understanding of several aspects, such as the relationship between the amplitude of the QRS wave and stroke volume in modulating cardiac output in sea, as there is considerable variability in this mechanism across different species and types of stressors.

Despite the potential of biologgers for assessing fish welfare in aquaculture environments, several limitations should be considered. The weight of the biologger should not exceed ≈ 2% of the fish’s body mass, which, in this study, limits its use to adult sea bass. Additionally, biologgers do not yet transmit real-time data wirelessly, meaning the logger must be removed from the fish to retrieve the data. However, biologgers remain a valuable tool for obtaining physiological measurements in captive studies, and with advancements in data transmission technologies, they could eventually be used to monitor fish welfare in large-scale aquaculture systems. Moreover, developing species- and life-stage-specific calibrations between acceleration, cardiac output and oxygen consumption would enhance the monitoring of fish behaviour and welfare in aquaculture settings.

## Conclusions

The implantation of biologger devices has been demonstrated to be an effective method for characterising the relationship between heart rate, acceleration and oxygen consumption. This has enabled the detection of changes in behavioural patterns, which are indicators of fish welfare status in aquaculture. Moreover, it has been demonstrated that heart rate can be employed as a reliable proxy to predict stress in European sea bass. The integration of cardiac activity data and acceleration levels with measurements of oxygen consumption and locomotion parameters provides a comprehensive understanding of sea bass behavioural and physiological responses, thereby paving the way for more reliable online monitoring of fish welfare in aquaculture.

## Materials and methods

### Ethical approval

Experimental protocols complied with the current laws of The Netherlands and were approved by the Dutch Central Committee for Animal Experiments (CCD), project number AVD40100202115078, 15 November 2021, and by the Animal Experiments Committee (DEC) and Authority for Animal Welfare (IvD) of Wageningen University and Research, experiment number 2021.D-0005.004, 7 July 2023. All methods are reported in accordance with ARRIVE guidelines.

### Experimental fish and conditions

European seabass individuals (*N* = 18) were reared at the animal experimental facilities of CARUS in Wageningen (The Netherlands) until reaching a suitable size for biologger implantation (*N* = 18; 391.1 ± 14.32 g). The fish were housed in a 600 L circular tank at 22.5 ± 0.45 °C, connected to a recirculating system, with 32.3 ± 1.8 ppt water and a photoperiod of 12:12. Water quality was checked biweekly for nitrogenous waste products. The fish were fed daily with pellets of commercial fish feed (Alltech Coppens B.V., Netherlands) at 1.4% of body mass. *N* = 12 fish were selected for surgical implantation of biologgers. The remaining *N* = 6 fish were considered as the control group.

### Heart rate and acceleration biologgers implantation

DST milli-HRT ACT (Star-Oddi, Gardabaer, Iceland) biologgers were implanted in 12 fish at size 423.03 ± 12.87 g body weight (BW) and 29.08 ± 0.49 cm standard length (SL). These loggers, hermetically sealed for biocompatibility, were 3.9 cm long and 1.3 cm wide and weighed 12 g. The surgical implantation of biologgers followed the protocol successfully applied by the staff in previous studies^16,17^. Fish were collected from the tank using a hand net and were anesthetized with 2-phenoxyethanol (0.3 ml.L^− 1^) in aerated water. When anesthetized, the fish was placed on a surgical table with the ventral side up, with a continuous flow of water with a low dose of 2-phenoxyethanol (0.15 ml.L^− 1^) passing over the gills. Between surgeries, the surgical equipment was rinsed in 70% ethanol and allowed to dry. Prior to the incision, 100 µL of lidocaine was injected at the incision area for pain relief. An incision of 1–2 cm was made with a scalpel on the ventral side at the edge of the pelvic fins, through which the disinfected logger was inserted in the abdominal cavity. The logger was positioned in the direction of the pericardial cavity and as close to it as possible, and then anchored with a twice-knotted single suture (Ethilon 3 − 0 669 H, Ethicon, NJ, USA; FS-1 24 mm 3/8c reverse cutting needle and 75 cm black monofilament non-absorbable suture). The incision was then stitched with two single sutures and betadine was applied to prevent infection. Surgery lasted for 11 min on average, and fish were swimming again after ~ 4 min from the surgery. After the logger implantation procedure, fish were allowed to recover in a quarantine tank for at least 11 days before the start of the first swimming trial. During recovery, they were gradually reintroduced to feeding: on the first day post-surgery, they received 20% of their normal ration, increasing to 40% on the second day, 60% on the third, 80% on the fourth, and reaching full feeding (1.4% of their biomass per day) from the fifth day onward. On the day of the swimming test, however, the selected fish were not fed.

### Swimming stress challenge

The swim challenge tests were performed in three 127 L Blažka-type swim-tunnels (see Van Den Thillart et al.^18^ for a detailed description). Tunnels were connected to a 400 L tank filled with seawater (22.0 ± 0.2 °C) and aerated to maintain high oxygen levels. Water from the tanks was recirculated through the tunnels using EHEIM pumps (2400 L.h^− 1^; EHEIM GmbH & Co. KG, Deizisau, Germany) and during oxygen measurements, the water inlet was closed by a valve.

Fish (control and tagged) were individually transferred from the tank to the swim tunnels and kept at rest for one hour, and where then swimming at incremental swimming speeds of 0.2, 0.4, 0.6, 0.8 and 1 m.s^− 1^ during one hour per speed (as in^41^. As shown in other species, an overnight acclimation period could contribute to increased stress before the start of the swim test. Thus, and to ensure comparability between species, the one-hour acclimation period was applied following previous studies^15,17,41^. During the last 10 min at each swimming speed, just before it was increased, tunnels were flushed for 10 min to re-establish high oxygen levels. Fish were allowed to acclimatize to a new swimming speed for 10 min before the valves were closed again and oxygen measurements commenced.

#### Respirometry

Each swim tunnel had a bypass with an oxygen probe in a respirometry system (DAQ-PAC-G4; Loligo Systems Aps, Tjele, Denmark) for measuring total oxygen content of the water in percentage, which allowed to assess oxygen consumption of the fish (in ΔO_2_%). Oxygen measurements were done for 40 min per swimming speed. In order to assess background (bacterial) oxygen consumption, several measurements were taken (without fish in the tunnels) at all flow speeds after swim challenge tests and extracted from the values measured with fish present.

The oxygen consumption (MO_2_ in mg O_2_.h^− 1^) was calculated from the decreasing oxygen contents when the valves were closed. The calculation was made following the formula:

$$M{O_2}=~\left( {\frac{{\Delta {O_2}\% \left( {DOmax*\frac{L}{{100}}} \right)}}{t}} \right)$$

Where DO_max_ is the maximum amount of oxygen dissolved in the water (7.157 mg O_2_ L^− 1^ at a temperature of 22 °C) and *L* is the volume of the tunnel (127 L) and *t* corresponds to the time in hours. The cost of transport (COT in O_2_.kg^− 1^.Km^− 1^) was calculated as $\:COT=\:\:MO2/\varDelta\:d$ where *Δd* is the covered distance in Km, estimated according to the flow speed and the duration of the swim challenge test.

Additionally, two more parameters characterizing swimming endurance were deduced: the optimal swimming speed (U_opt_) and the minimum cost of transport (*COT*_*min*_). *U*_*opt*_ is defined as the flow speed at which fish swim most efficient and the cost of transport (*COT*) reaches its minimum (in m.s^− 1^ or bl.s^− 1^). *COT*_*min*_ corresponds to the *COT* value at *U*_*opt*_ (in mg O_2_ Kg^− 1^.Km^− 1^). These parameters are determined by plotting a polynomial trend line of *COT* against water flow speed for each individual. The point on this trend line with the lowest COT (*COT*_*min*_) was calculated by equalling the first derivative to zero^43^.

#### Locomotory behaviour

Seabass locomotory behaviour during the swimming stress challenge test was filmed with Basler USB 3.0 cameras (ace acA2040-90 μm NIR) at a frame rate of 75 fps and at 7.5 ms of exposure time. Pixels were binned 2 × 2 to improve sensitivity by a factor of 4. Cameras were filming from below the tunnels and a white translucid background was placed above the tunnels to create a homogeneous light background. The cameras’ vision field covered the full width of the tunnel and the full length excluding the last ~ 20 cm downstream. Custom software developed in Python was used to detect and save the fish contour in real-time. To detect the fish, a luminance threshold selected dark objects relative to the light background. The fish were selected among the detected objects (using the find_contours routine) based on size (surface area) and length/width ratio of the contour. See Arechavala-López et al.^3^ for further details on the central axis tracking and detection. The quantification of tail beat parameters was done by selecting a point in the tail at about 0.8 x Standard Length (SL) of the fish and by determining the lateral excursion relative to the midline through the head. Tail beat frequency (TBF) and amplitude (TBA) were obtained by performing a spectral analysis on the tail excursion as a function of time. Spectrograms were calculated with a temporal window size of 0.85 s, shifted frame by frame. Frequency and amplitude were determined based on the maximum in the spectrogram at each frame. A similar calculation was performed on the width of the head, at the location of the opercula, to obtain frequency (HWF) and amplitude (HWA). These motion parameters reflect the rhythmic movements of the opercula, which can be different from swimming frequencies.

### Crowding stress challenge

The crowding stress challenge test was performed on the tagged individuals (*N* = 12) four days after completing the swimming stress challenge tests. The housing tank (600 L circular tank) was filled with ~ 200 L of water. The test followed the protocol developed by Svendsen et al.^12^, which consisted in four steps: reducing the water level to a point at which the dorsal fin was exposed to air and then (1) refilling immediately; (2) refilling after 1 min; (3) refilling after 5 min; and (4) chasing the fish with a net during 5 min and then refilling. Each iteration was executed on the hour (at 10, 11, 12 and 13 h), and biologgers monitored HR and external acceleration during the whole challenge and until four hours after the test finished. Measurements were taken every 10 min and categorized as: *stress* (1 to 4) that corresponds to each moment of crowding stress; *R* (1 to 4) for the measurements taken during the 50 min following each stressor; and *final rec* that corresponds to the values obtained two to four hours after the end of the crowding stress challenge.

### Biologgers’ data processing

Loggers were recovered and data downloaded after experimentation. Heart rate (HR) and amplitude of the heart signal (AMP) was derived by manual annotation from the electrocardiogram (ECG) signal at 200 Hz, recorded every 10 min for 7.5 s as beats per minute (bpm) using HRT-Analyzer v.1.3.1 software from Star-Oddi and a custom filter file for quality improvement. For each swimming speed, six heart rate measurements were obtained, and all were used to set the heart rate for that particular swimming speed. Acceleration (ACC) was measured as external acceleration, which is the recorded three-axis acceleration above standard gravity defined with static calibration, normalized, and calculated as the vectorial sum in milli-g (more detailed description in Rouyer et al.^44)^. Reported is the average external acceleration value (ACC, which is an average of 600 3-axis measurements over 1 min), recorded at 10 Hz. The baseline values of heart rate, amplitude and external acceleration were calculated by averaging 20 measurement points throughout the days before the experiments.

### Data analysis

Differences in biometry (i.e. SL and BW) and estimated swimming performance parameters (i.e. U_opt_ and COT_min_) between tagged and non-tagged fish were evaluated through analyses of variance (ANOVA). In cases where the assumptions of the model (homogeneity of variances and normality of the residuals) were not met, non-parametric Kruskal-Wallis tests were performed. Linear models were fitted to assess the relationship between water flow speed and dependent variables (i.e. MO2, COT, TBF, HWF, TBA and HWA) in both groups (tagged and non-tagged individuals). To evaluate differences between groups regarding the dependent variables, an ANOVA was conducted on the fitted models. Linear mixed models (LMM) were fitted to assess differences in external acceleration (ACC), heart rate (HR) and amplitude (AMP) across swimming speeds (fixed effect), accounting for individual variability as random effect in all the models. To further explore pairwise differences in ACC, HR and AMP between flow speeds, Tukey’s Honest Significant Differences (HSD) post-hoc test was applied. Additionally, a correlation matrix was calculated for the dependent variables and a correlation plot was generated. Simple regressions were applied to assess the relationship between MO_2_ and TBF with ACC, HR and AMP during the swimming stress challenge.

To evaluate the effects of the crowding stress challenge test in cardiac activity and acceleration, LMMs were applied considering HR, ACC or AMP as response variables, the test phase as a fixed effect, and the individual as a random effect in order to account for individual variability. Additionally, a post-hoc Tukey HSD test was performed to determine differences across the different crowding stress challenge stages.

All the statistical analyses were performed using the R software^45^, version 4.3.2. Plots were created with the ggplot2^46^ and corrplot^47^ packages. The nlme^48^ and lme4^49^ packages were employed to fit linear mixed models. The multcomp^50^ package was used to perform the Tukey HSD post-hoc test. Results were considered significant at *p* < 0.05. All values are reported as means ± SE.

## Data Availability

The datasets generated and analysed during the current study are available from the corresponding author on reasonable request.
